# Suppression of PPARγ-mediated monoacylglycerol *O*-acyltransferase 1 expression ameliorates alcoholic hepatic steatosis

**DOI:** 10.1038/srep29352

**Published:** 2016-07-11

**Authors:** Jung Hwan Yu, Su Jin Song, Ara Kim, Yoonjeong Choi, Jo Woon Seok, Hyo Jung Kim, Yoo Jeong Lee, Kwan Sik Lee, Jae-woo Kim

**Affiliations:** 1 Department of Biochemistry and Molecular Biology, Integrated Genomic Research Center for Metabolic Regulation, Institute of Genetic Science, Yonsei University College of Medicine, Seoul 120-752, Korea; 2 Brain Korea 21 PLUS Project for Medical Science, Yonsei University, Seoul 120-752, Korea; 3 Division of Metabolic Disease, Center for Biomedical Sciences, National Institutes of Health, Cheongwon-gun, Chungbuk 363-951, Korea; 4 Department of Internal Medicine, Yonsei University College of Medicine, Seoul 120-752, Korea; 5 Severance Biomedical Science Institute, Yonsei University College of Medicine, Seoul 120-752, Korea

## Abstract

Alcohol consumption is one of the major causes of hepatic steatosis, fibrosis, cirrhosis, and superimposed hepatocellular carcinoma. Ethanol metabolism alters the NAD^+^/NADH ratio, thereby suppressing the activity of sirtuin family proteins, which may affect lipid metabolism in liver cells. However, it is not clear how long-term ingestion of ethanol eventually causes lipid accumulation in liver. Here, we demonstrate that chronic ethanol ingestion activates peroxisome proliferator-activated receptor γ (PPARγ) and its target gene, monoacylglycerol O-acyltransferase 1 (MGAT1). During ethanol metabolism, a low NAD^+^/NADH ratio repressed NAD-dependent deacetylase sirtuin 1 (SIRT1) activity, concomitantly resulting in increased acetylated PPARγ with high transcriptional activity. Accordingly, SIRT1 transgenic mice exhibited a low level of acetylated PPARγ and were protected from hepatic steatosis driven by alcohol or PPARγ2 overexpression, suggesting that ethanol metabolism causes lipid accumulation through activation of PPARγ through acetylation. Among the genes induced by PPARγ upon alcohol consumption, MGAT1 has been shown to be involved in triglyceride synthesis. Thus, we tested the effect of MGAT1 knockdown in mice following ethanol consumption, and found a significant reduction in alcohol-induced hepatic lipid accumulation. These results suggest that MGAT1 may afford a promising approach to the treatment of fatty liver disease.

Alcoholic liver disease, which is a major cause of morbidity and mortality worldwide, is associated with increased cardiovascular disease and diabetes^1^. Accumulation of fat in the liver in response to alcohol consumption can lead to more harmful forms of liver disease such as fibrosis, cirrhosis, and end-stage liver injury. Despite numerous studies on the pathogenesis of alcoholic liver diseases, targeted therapies based on the mechanism by which alcohol consumption causes hepatic steatosis are unavailable. The spectrum of alcoholic liver disease ranges from simple steatosis to more serious injury including cirrhosis. Because fatty liver damage is reversible when detected early, the best way to resolve this alcohol-mediated liver damage at this stage is abstaining from alcohol^2^. However, in order to provide improved, more effective therapies, elucidation of the mechanism by which ethanol metabolism causes lipid accumulation in the liver is required.

Recent studies indicate that ethanol increases fatty acid synthesis in hepatocytes by regulating lipid metabolism-associated transcription factors such as sterol regulatory element-binding protein 1c (SREBP1c) and carbohydrate-responsive element-binding protein (ChREBP), which promote fatty acid synthesis via up-regulation of lipogenic genes^3^. While ethanol is metabolized in liver, alcohol dehydrogenase and aldehydrogenase catalyze the conversion of NAD^+^ to NADH, thereby reducing the NAD^+^/NADH ratio similar to the fed state, in which the glycolysis pathway is activated and, in turn, sirtuin 1 (SIRT1) is inactivated^4^. SIRT1 is an NAD-dependent deacetylase that is activated in response to fasting, caloric restriction, and physical exercise^5^. This enzyme plays an important role in hepatic lipid metabolism by modulating the acetylation of transcription factors such as peroxisome proliferator-activated receptor gamma coactivator 1-α (PGC-1α)^6^. Recent research reveals that SIRT1 signaling is also associated with alcoholic liver disease as SIRT1 stimulation protects against alcohol-induced liver damage^6^,^7^. Because ethanol metabolism mimics the fed state even in the absence of glucose in terms of the NAD^+^/NADH ratio, these studies suggest that ethanol metabolism affects lipid metabolism through SIRT1 activity.

In adipocytes, SIRT1 represses peroxisome proliferator-activated receptor γ (PPARγ) activity by recruiting a co-repressor, which results in fat mobilization^8^. Others have reported that SIRT1 directly deacetylates PPARγ, promoting brown remodeling of white adipocytes^9^. Thus, one of the mechanisms by which SIRT1 modulates hepatic lipid metabolism may involve PPARγ, a master regulator of lipid metabolism^10^,^11^. Among the two isoforms, PPARγ2 is mainly present in adipose tissue, intestines, and macrophages to regulate fatty acid storage and glucose metabolism. In normal liver, PPARγ expression remains low. However, its expression is increased in a mouse model of obesity and plays a critical role in hepatic steatosis by regulating the expression of lipogenic genes^12^. In addition, hepatic PPARγ expression is associated with triglyceride (TG) synthesis and lipid accumulation^13^,^14^. Recently, we reported that increased PPARγ2 expression is a major contributor to high-fat-diet-induced hepatic steatosis, demonstrating that monoacylglycerol O-acyltransferase 1 (MGAT1), a PPARγ-regulated enzyme, plays a critical role in lipid accumulation^15^.

MGAT1 is an enzyme that catalyzes the synthesis of diacylglycerol from monoacylglycerol and fatty acyl CoA. Thus, MGAT1 contributes to lipid accumulation via an alternative pathway for TG synthesis^16^. MGAT1, along with PPARγ, is expressed at low levels in normal liver but is highly up-regulated in diet-induced hepatic steatosis^15^. In this regard, inhibiting MGAT1 expression eventually suppressed hepatic lipid accumulation, as demonstrated by several studies in which MGAT1 is knocked down by adenovirus-mediated small hairpin RNA (shRNA)^15^, antisense oligonucleotides^17^, and liver-specific non-viral small interfering RNA (siRNA)^18^. Although these studies are restricted to non-alcoholic hepatic steatosis, we hypothesize that PPARγ and its downstream effector MGAT1 may play a role in alcohol-induced hepatic lipid accumulation if SIRT1 affects hepatic PPARγ during ethanol metabolism.

In this study, we found that PPARγ is activated in alcohol-induced hepatic steatosis and associated with reducing SIRT1 activity upon ethanol metabolism. Furthermore, our data suggest that inhibition of MGAT1 efficiently attenuated lipid accumulation due to alcohol consumption in liver. Therefore, we suggest that PPARγ is an important regulator of ethanol-induced hepatic steatosis, and that the development of an MGAT1 inhibitor could be an effective therapy for treating alcoholic or non-alcoholic hepatic steatosis.

## Results

### Ethanol decreased the NAD^+^/NADH ratio and SIRT1 activity

**Figure 1:**
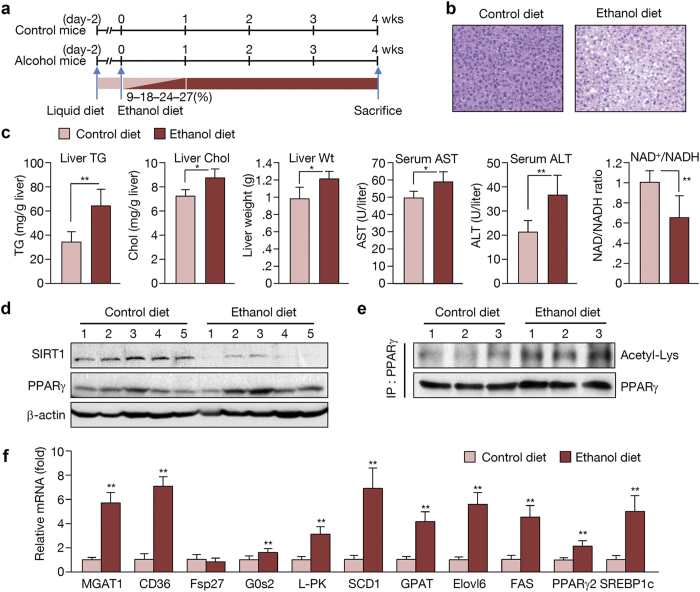
Ethanol decreases the NAD^+^/NADH ratio and SIRT1 activity to cause PPARγ acetylation. (**a**) Schedule of ethanol and control diet regimens. (**b**) H&E staining performed on liver sections from mice. (**c**) Hepatic TG and cholesterol content, liver weight, serum aspartate transaminase (AST) alanine transaminase (ALT) levels, and NAD^+^/NADH ratio in the livers of control diet and ethanol diet-fed mice. (n = 6 per group) (**d**) Western blot analysis of SIRT1, PPARγ, and β-actin. (n = 5 per group) (**e**) PPARγ acetylation in control and ethanol diet-fed mice. (n = 3 per group) (**f**) Expression of PPARγ and SREBP1c and their target genes in control and ethanol diet-fed mice. (n = 6–8 per group) Data represent the mean ± SD. *P < 0.05, **P < 0.01.

To study the effects of alcohol metabolism, we used C57BL/6 (B6) mice, which are widely used in metabolic disease research. Male B6 mice were pair-fed a Liber-Decarli liquid diet with or without (27% of total calories) ethanol for 4 weeks (Fig. 1a). Exposure to an ethanol diet for 4 weeks resulted in a significant increase in hepatic TG content and liver weight, indicating that ethanol-induced hepatic steatosis and increased liver weight (Fig. 1b,c; oil-red-O staining shown in Supplementary Fig. S1). Notably, the NAD^+^/NADH ratio was significantly decreased in ethanol-fed mice (Fig. 1c). The level of SIRT1, an NAD-dependent deacetylase, also decreased, while PPARγ was not remarkably changed (Fig. 1d). However, acetylation of PPARγ clearly increased in ethanol-fed mice (Fig. 1e), suggesting that the SIRT1 activity required to deacetylate PPARγ is suppressed upon ethanol feeding. Increased PPARγ acetylation resulted in marked up-regulation of its target genes, including MGAT1, CD36, and G0s2, while PPARγ mRNA increased slightly (Fig. 1f). Another transcription factor known to play an important role in the pathophysiology of alcoholic hepatic steatosis, SREBP1c, was also highly expressed along with its target genes, which include liver-type pyruvate kinase (L-PK), stearyl CoA desaturase 1 (SCD1), glycerol phosphate acyltransferase (GPAT), fatty acid elongase 6 (Elvol6), and fatty acid synthase (FAS) (Fig. 1f). These data indicate that the fatty acid *de novo* synthesis pathway is also elevated following alcohol ingestion.

The mRNA levels of the seven known types of sirtuins revealed that expression of all sirtuin types was reduced in the alcohol-fed group (Supplementary Fig. S2). Of these, we checked the relationship between SIRT1 and ethanol-induced lipid metabolism, mainly focused on PPARγ activity. Further studies may be required to explore the linkage between alcohol metabolism and other sirtuins such as sirtuin 3 and 6, which are known to be involved in body metabolism.

### SIRT1 protects the liver from alcohol-induced hepatic steatosis

**Figure 2:**
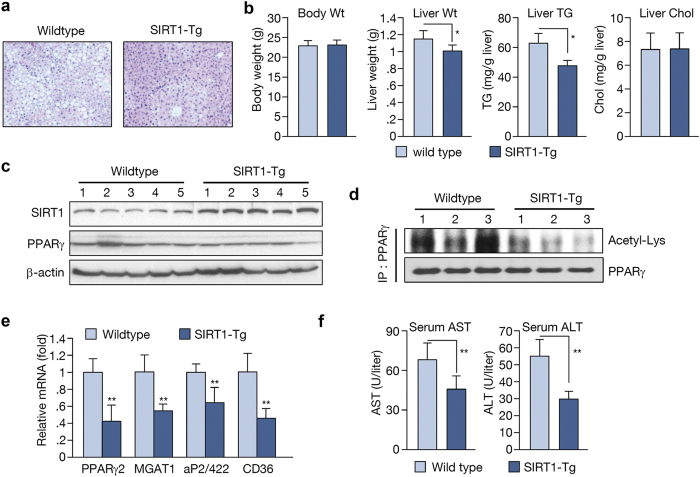
SIRT1 transgenic mice exert a protective effect on alcohol-induced hepatic steatosis. (**a**) H&E staining performed on liver sections from mice. (**b**) Body weight, liver weight, hepatic TG, and cholesterol in wild type and SIRT1 transgenic mice. (**c**) Western blot analysis of SIRT1, PPARγ, and β-actin. (n = 5 per group) (**d**) PPARγ acetylation in wild type and SIRT1 transgenic mice. (n = 3 per group) (**e**) Real-time PCR analysis of PPARγ and its target genes in liver. (**f**) AST and ALT levels in blood samples. (n = 6 per group). Data represent the mean ± SD. *P < 0.05, **P < 0.01.

Next, we used SIRT1 transgenic mice to evaluate its role in ethanol metabolism. Wildtype and SIRT1 transgenic mice were pair-fed a Liber-Decarli liquid diet with ethanol (27% of total calories) for 4 weeks. While wildtype mice developed a significant accumulation of lipid in the liver, SIRT1 transgenic mice displayed a protective effect on ethanol-induced hepatic steatosis (Fig. 2a; oil-red-O staining shown in Supplementary Fig. S1). Throughout the experimental period, food intake was similar and ethanol feeding had no apparent effect on the health status of both mouse groups. Although body weight remained unchanged, the liver weights were lower in SIRT1 transgenic mice (Fig. 2b). Liver TG content in these mice was also decreased significantly compared to wildtype animals (Fig. 2b). The level of PPARγ protein did not differ significantly in SIRT1 transgenic mice (Fig. 2c), but acetylation of PPARγ appeared to decrease (Fig. 2d). Furthermore, the expression of PPARγ and its target genes, including MGAT1, aP2/422, and CD36, was also reduced (Fig. 2e). These data suggest that PPARγ activity, with subsequent PPARγ expression, also decreased. The levels of aspartate transaminase (AST) and alanine transaminase (ALT), which are indicators of liver inflammation, were also decreased in SIRT1 transgenic mice (Fig. 2f).

**Figure 3:**
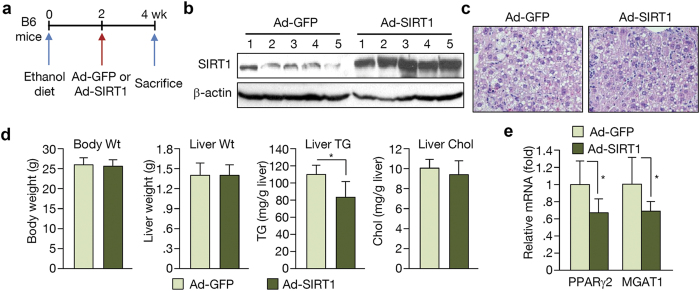
The effect of SIRT1 overexpression via adenovirus injection on alcohol-induced hepatic steatosis. (**a**) Schedule of SIRT1 adenovirus injection. Ad-SIRT1 or Ad-GFP was introduced via tail vein injection at 2 weeks after initiation of ethanol feeding. The mice were sacrificed 2 weeks later. (**b**) Western blot analysis showing SIRT1 expression in the liver of Ad-GFP- and Ad-SIRT1-injected mice. (**c**) H&E staining performed on liver sections from mice. (**d**) Body weight, liver weight, hepatic TG, and cholesterol content. (**e**) Real-time PCR analysis of PPARγ and MGAT1 expression. (n = 5) Data represent the mean ± SD. *P < 0.05.

Because these transgenic mice express SIRT1 throughout the body, we could not rule out effects from other organs such as adipose tissue. To examine the effect of SIRT1 on hepatic lipid accumulation directly, we employed adenoviruses as vectors to express SIRT1 because they preferentially target the liver in mice^19^. During ethanol feeding, adenovirus overexpressing GFP or SIRT1 was injected into the tail vein 2 weeks before the end of the experimental period (Fig. 3a). This administration led to robust expression of hepatic SIRT1 (Fig. 3b), which attenuated ethanol-induced hepatic lipid accumulation (Fig. 3c,d). Interestingly, liver weight was similar with this short-term expression of SIRT1 (Fig. 3d). SIRT1 overexpression in the liver caused down-regulation of PPARγ and its target gene, MGAT1 (Fig. 3e), suggesting that ethanol metabolism eventually affects lipid metabolism through SIRT1. Taken together, these data indicate that SIRT1 has a protective and beneficial role in alcohol-induced hepatic steatosis.

### SIRT1 attenuates PPARγ2-induced hepatic steatosis

**Figure 4:**
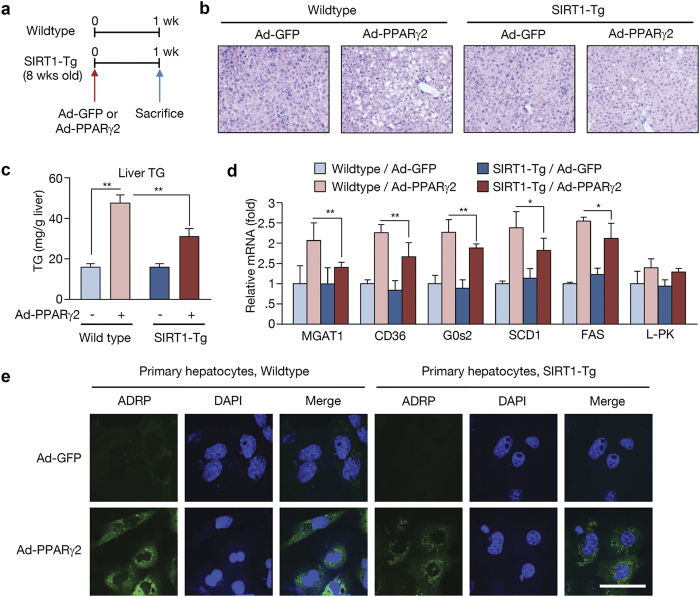
The effect of PPARγ2 overexpression on wild type and SIRT1 transgenic mice. (**a**) Schedule of PPARγ2 overexpression. Wild type and SIRT1 transgenic mice were injected with Ad-PPARγ2 or an Ad-GFP via the tail vein, fed a chow diet for 1 week, and then sacrificed. (**b**) H&E staining performed on liver sections of wild type and SIRT1 transgenic mice. (**c**) Hepatic TG content in wild type and SIRT1 transgenic mice infected with Ad-GFP or Ad-PPARγ2. (**d**) Real-time PCR analysis of PPARγ target genes and lipogenic genes in wild type and SIRT1 transgenic mice. (**e**) Immunofluorescence staining for adipose differentiation-related protein (ADRP, green) in mouse primary hepatocytes. Nuclei were stained with DAPI and fluorescence was visualized by confocal microscopy. Scale bar = 50 μm. Data in c and d represent the mean ± SD. *P < 0.05, **P < 0.01.

We demonstrated that SIRT1 overexpression attenuates ethanol-induced hepatic steatosis, in which PPARγ acetylation is decreased. To verify the involvement of PPARγ in SIRT1-mediated effects on the fatty liver, we administered adenoviral PPARγ2 (Ad-PPARγ2) into wildtype or SIRT1 transgenic mice via tail vein injection (Fig. 4a). As reported previously^15^, Ad-PPARγ2 injection produced severe hepatic steatosis (Fig. 4b; oil-red-O staining shown in Supplementary Fig. S1) with higher levels of hepatic TG than control Ad-GFP mice (Fig. 4c). Histological analysis revealed the presence of numerous fat droplets in the liver of Ad-PPARγ2-injected mice. However, SIRT1 transgenic mice overexpressing PPARγ2 exhibited lower levels of hepatic TG than wildtype mice (Fig. 4b,c). Ad-PPARγ2 injection induced the expression of several PPARγ targets and lipogenic genes. However, this increase in gene expression was attenuated in SIRT1 transgenic mice (Fig. 4d).

We also analyzed the effect of PPARγ2 overexpression in primary mouse hepatocytes. These cells were subjected to immunocytochemistry to detect adipose differentiation-related protein (ADRP), which is a target gene of PPARγ that localizes to the lipid droplet. PPARγ2 overexpression resulted in increased ADRP expression in primary hepatocytes; however, this increase was significantly attenuated in cells isolated from SIRT1 transgenic mice (Fig. 4e). These results suggest that PPARγ acts as a major regulator in hepatic TG synthesis and SIRT1 inhibits PPARγ-induced hepatic steatosis.

### MGAT1 knockdown protects against alcohol-induced hepatic steatosis

**Figure 5:**
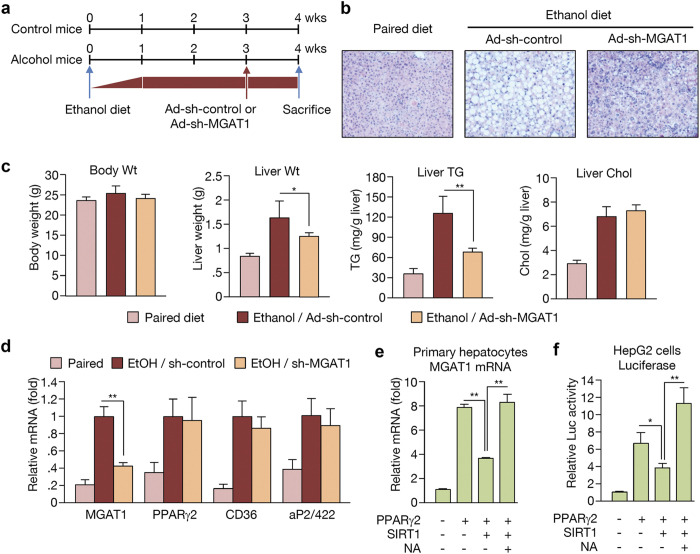
MGAT1 knockdown suppresses alcohol-induced hepatic steatosis. (**a**) Schedule of MGAT1 knockdown in ethanol-fed mice. Adenoviral sh-control or sh-MGAT1 was introduced via tail vein injection at 3 weeks after initiation of ethanol feeding. One week later, the mice were sacrificed. (**b**) H&E staining performed on liver sections from mice in paired, ethanol-fed with sh-control, and ethanol-fed with sh-MGAT1 groups. (**c**) Body weight, liver weight, hepatic TG, and cholesterol content. (**d**) Real-time PCR analysis showing the expression of PPARγ and its target genes. (n = 6). Data represent the mean ± SD. *P < 0.05. (**e**) Primary hepatocytes (4 × 10^5^ cells per well) cultured on 6-well plates were infected with 50 MOI of Ad-GFP, Ad-PPARγ2, and Ad-SIRT1 with or without nicotinamide (NA, 10 μM). MGAT1 mRNA was analyzed by real-time PCR. (**f**) Luciferase assay using human MGAT1 promoters (~2 kb) was performed. In HepG2 cells, MGAT1 promoter constructs were co-transfected with or without PPARγ2/RXRα overexpression vectors along with 100 MOI of either Ad-GFP or Ad-SIRT1. Data represent the mean ± SD from three independent experiments performed in duplicate. *P < 0.05, **P < 0.01.

Previously, we reported that knockdown of PPARγ-regulated MGAT1 expression can successfully improve diet-induced non-alcoholic hepatic steatosis in murine and human models^15^,^20^. Our results shown here strongly suggest that the PPARγ-MGAT1 axis contributes to the development and aggravation of lipid accumulation both in non-alcoholic and alcoholic hepatic steatosis. However, the mechanism of PPARγ activation might be different. To examine whether MGAT1 suppression can reduce ethanol-induced hepatic steatosis, male B6 mice were fed a Liber-Decarli liquid diet with or without ethanol (27% of total calories) for 3 weeks and then administered with adenoviral sh-control or sh-MGAT1 via tail vein injection. After 1 week of continued diet, mice were sacrificed for analysis (Fig. 5a). As shown in Fig. 5b (oil-red-O staining shown in Supplementary Fig. S1), knockdown of hepatic MGAT1 significantly improved ethanol-induced hepatic steatosis. The hepatic TG level was reduced by 42% after only 1 week of MGAT1 knockdown (Fig. 5c). Furthermore, hepatic MGAT1 knockdown also resulted in decreased liver weight, suggesting that MGAT1 expression plays an important role in alcohol-induced hepatic steatosis (Fig. 5c). Targeting MGAT1 did not affect the expression of PPARγ and its other regulatory genes such as aP2/422 and CD36, suggesting that MGAT1 is a critical factor involved in the accumulation of hepatic lipid upon ethanol feeding (Fig. 5d).

To verify the role of the SIRT1-PPARγ-MGAT1 axis in this process, we tested MGAT1 mRNA expression in the presence of PPARγ and/or SIRT1. As shown in Fig. 5e, SIRT1 efficiently suppressed PPARγ2-induced MGAT1 expression. Nicotinamide (NA), a known SIRT1 inhibitor, counteracted the suppressive effects of SIRT1. Luciferase assays also showed that SIRT1 suppresses PPARγ2-induced MGAT1 promoter activity in human hepatoma-derived HepG2 cells (Fig. 5f). To evaluate whether SREBP1c, another major transcription regulator of hepatic steatosis, regulates MGAT expression, we overexpressed SREBP1c into human HepG2 cells. Our data show that SREBP1c did not affect the expression level of all three MGAT subtypes in human cells (Supplementary Fig. S3), suggesting that increased SREBP1c and PPARγ during ethanol feeding contributes to lipid accumulation via distinct mechanisms.

A previous study reported that PPARγ acetylation sites (lysine residues 98, 107, 218, 268, and 293) were affected by SIRT1^9^. Therefore, we generated various PPARγ2 mutant forms (K98R, K107R, K218R, K268R, and K293R) to evaluate the role of the acetylation sites in modulating PPARγ-induced MGAT1 expression. However, as shown in Supplementary Fig. S4, we could not find a significant difference in PPARγ activity following mutation of the acetylation sites, which regulate MGAT1 expression. This result suggests that MGAT1 expression is regulated by acetylation on other sites or combination of various acetylation.

### Ethanol metabolism in binge drinking

**Figure 6:**
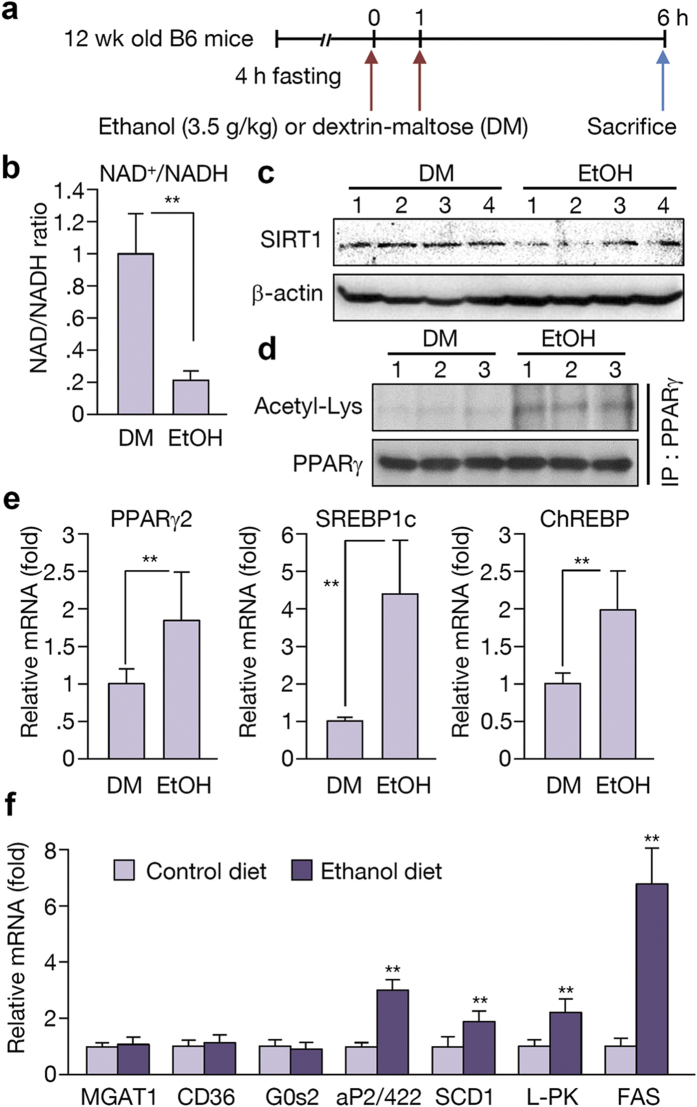
Ethanol metabolism in binge drinking leads to the activation of SREBP1c and ChREBP. (**a**) Schedule of binge drinking experiment. B6 mice were fasted for 4 h before receiving two gavages of equivalent calories of ethanol at 3.5 g/kg or dextrin-maltose (DM). Mice were sacrificed 6 h later. (**b**) NAD^+^/NADH ratio examined in DM and ethanol-fed mice group. (n = 6 per group) (**c**) Western blot analysis showing SIRT1 expression in DM and ethanol-fed mice group. (n = 4 per group) (**d**) PPARγ acetylation in DM and ethanol-fed mice group. (n = 3 per group) (**e,f**) Real-time PCR analysis showing expression of PPARγ, SREBP1c, ChREBP, and their target genes. (n = 6). Data represent the mean ± SD. **P < 0.01.

**Figure 7:**
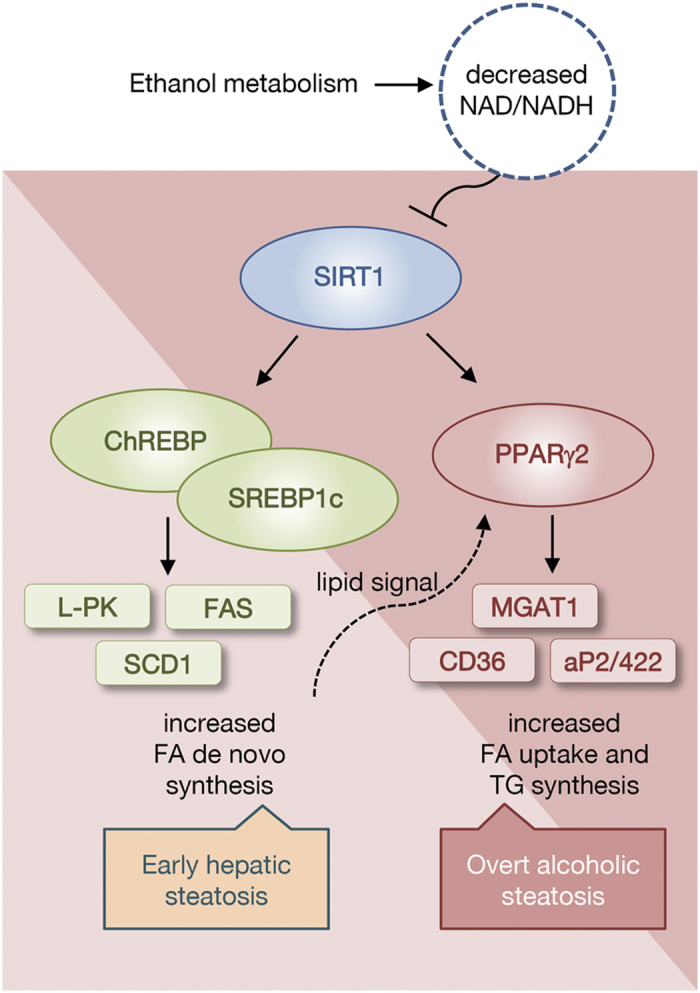
Model of alcohol-induced hepatic steatosis. During ethanol digestion, NAD^+^ is converted into NADH by alcohol dehydrogenase and aldehyde dehydrogenase. A low NAD^+^/NADH ratio represses NAD-dependent deacetylase sirtuin 1 (SIRT1) activity. Reduced SIRT1 activity causes the activation of SREBP1c, ChREBP, and PPARγ. In the early phase, expression of SREBP1c and ChREBP target genes, such as L-PK, FAS, and SCD1, are increased, and then PPARγ target genes including MGAT1 are increased. Expression of PPARγ and its target genes lead to the development of alcoholic steatosis.

Binge drinking is the act of consuming heavy amounts of alcohol in a short time period. Recently, the role of ChREBP in binge drinking was reported^3^. In this report, acute alcohol consumption affected the activity of ChREBP by regulating its acetylation. Based on this finding, we explored the role of PPARγ and the early response to alcohol consumption through a binge drinking mouse model. Twelve-week-old male C57BL/6J mice were fasted for 4 h before receiving two doses of gavages with equivalent calories of DM or ethanol at 3.5 g/kg (Fig. 6a). Examination of these animals revealed that the NAD^+^/NADH ratio was decreased in the binge drinking group compared to the DM group (Fig. 6b). The SIRT1 level was also decreased after binge drinking (Fig. 6c). PPARγ expression and its acetylation were increased with binge drinking (Fig. 6d,e). Also, the level of SREBP1c and ChREBP mRNA was increased in the livers of the ethanol-consuming group (Fig. 6e). Accordingly, the expression of SREBP1c and ChREBP target genes such as SCD1, L-PK, and FAS increased; however, expression of PPARγ target genes remained at a low level (Fig. 6f). These results suggest that the expression level of PPARγ in this setting was not sufficient to induce its target genes even though PPARγ mRNA was up-regulated by two-fold. Thus, we hypothesize that binge drinking initially affects SREBP1c or ChREBP to develop a mild steatosis primarily by fatty acid *de novo* synthesis, which later induces a sufficient level of acetylated PPARγ and its target genes, and ultimately aggravating hepatic steatosis (Fig. 7).

## Discussion

Excessive alcohol consumption is an important public health problem that contributes substantially to the global burden of mortality and morbidity. In the 2010 Global Burden of Disease study, alcohol-attributable liver disease was responsible for 493,300 deaths (156,900 females and 336,400 males) representing 0.9% of all global deaths^21^. Therefore, efforts to reduce alcohol consumption and prevent alcohol-induced hepatic damage are needed. Alcoholic liver disease starts with hepatic steatosis, which is characterized by an increase in intrahepatic TG. Continuous alcohol consumption leads to hepatic inflammation and liver fibrosis. Studies estimate that a third of patients with steatosis will develop hepatic inflammation and 8% to 20% of patients with steatosis will eventually progress to cirrhosis^1^. The main causes for alcoholic fatty liver are known to be accumulation of acetaldehyde, tumor necrosis factor α, endoplasmic reticulum stress, 2-arachidonoylgycerol, and adenosine, which increase SREBP1c activity and induce fatty acid synthesis, leading to alcoholic fatty liver. Other transcription factors also contribute to the pathogenesis of alcoholic liver disease. For example, PPARα, another major regulator, decreases fatty acid β-oxidation^1^. Recently, ChREBP was reported to play a role during the early period of alcohol consumption^3^. On the other hand, hepatic PPARγ, which is known as a critical regulator of TG accumulation in adipose tissue, has yet to be investigated in alcohol-induced hepatic steatosis until now.

A ligand-activated transcription factor, PPARγ is a member of the nuclear receptor superfamily that primarily regulates adipogenesis and lipid accumulation^22^. Because PPARγ expression in the liver is low compared to that in adipose tissue, the role of this transcription factor in the liver remains controversial^13^. In the past decade, studies found that hepatic PPARγ is significantly increased in an obese animal model and plays an important role in fatty liver formation^23^,^24^. In our study, alcohol-fed mice appeared to exhibit an increase in hepatic PPARγ protein to a certain extent; however, comparison to control mice nullified any apparent increase. However, the expression of PPARγ target genes increased in alcohol-fed mice. Therefore, we investigated the post-translational modification of PPARγ to examine how ethanol regulates PPARγ activity. Studies have demonstrated that PPARγ function is regulated by several post-translational modifications, including phosphorylation, acetylation, sumoylation, and ubiquitination^24^,^25^. PPARγ is phosphorylated within the AF1 region by mitogen-activated protein kinases or cyclin-dependent kinases (Cdk7 and Cdk9), which regulate PPARγ activity in opposing manners^26^. Moreover, it was found that PPARγ is phosphorylated within the ligand binding domain at Ser273 by Cdk5 to control the expression of a distinct group of genes that becomes deregulated in obesity^27^. Sumoylation of PPARγ in the AF1 region represses its transcriptional activity, possibly by recruiting a co-repressor^28^. Additionally, PPARγ has been shown to be ubiquitinated, which is enhanced by ligand binding like thiazolidinediones^29^. The regulation of PPARγ function by acetylation has not been extensively studied; however, it is evident that PPARγ activity is associated with acetylation as PPARγ activity is attenuated by the presence of a deacetylase such as HDAC3 and SIRT1^30^.

SIRT1 regulates lipid metabolism by deacetylating lysine residues on transcription factors such as SREBP1c and PGC1α in liver^31^,^32^. However, the mechanism by which SIRT1 regulates hepatic PPARγ function, especially in alcoholic fatty liver, remains poorly understood. In this study, we established that SIRT1 is involved in alcoholic hepatic steatosis by regulating the acetylation of PPARγ and its activity. We also observed that PPARγ overexpression could induce hepatic steatosis, and that knockdown of MGAT1, a PPARγ target gene, could inhibit hepatic lipid accumulation. We previously showed that MGAT1 is a direct target gene of PPARγ, by identifying functional PPAR-response element in the promoter^15^, and also reported that human MGAT1 is also regulated by PPARγ shown by luciferase assay^20^. Therefore, we propose that the PPARγ signaling pathway is critical in hepatic lipid synthesis and fatty liver formation. In order to examine whether other pathways may involve in the regulation of MGAT1 in ethanol feeding, we also investigated SREBP1c, which is reported to play a role in alcoholic hepatic steatosis. Supplementary Fig. S3, however, showed that the expression of MGAT genes did not change in response to SREBP1c overexpression. In addition, it is noteworthy that MGAT1 was not induced upon binge drinking shown in Fig. 6, where SREBP1c and ChREBP are highly expressed. Thus, it is unlikely that SREBP1c or ChREBP regulates MGAT1. Nevertheless, it is still possible that other pathways in ethanol feeding regulate MGAT1, because the short-term binge drinking could not activate the MGAT1 transcription even in the induction of PPARγ and its acetylation (Fig. 6). It may be due to the lack of lipid signaling required for PPARγ transcriptional activity, but we cannot rule out the involvement of other pathways.

The causes of hepatic steatosis have been categorized as either alcoholic or non-alcoholic. Despite this, it is difficult to differentiate between these types of hepatic steatosis in the clinic. Furthermore, alcohol synergistically increases the prevalence and severity of hepatic steatosis in obese patients^33^,^34^. Discovering similar pathways between non-alcoholic and alcoholic hepatic steatosis is a useful strategy for developing effective therapeutics for treating hepatic steatosis. In this study, we identified that the SIRT1-PPARγ-MGAT1 axis is critically involved in alcoholic hepatic steatosis, making MGAT1 a potential therapeutic target of hepatic fatty liver disease.

## Materials and Methods

### Mice and diet

Male C57BL/6J mice were purchased from SLC (Japan). The animals were maintained in a temperature-controlled room (22 °C) on a 12:12-h light–dark cycle. Mice (at 7 or 8 weeks of age) were fed a Lieber-DeCarli liquid diet (Dyets) containing 1 Kcal/ml, of which 18% was derived from protein, 35% of fat, and either 47% from carbohydrate (control diet) or 20% from carbohydrate and 27% from ethanol (alcohol diet) for up to 4 weeks. Ethanol was introduced gradually by increasing the content by 9% of its total caloric-intake until the mice were consuming a diet containing 27% ethanol, which was then continued for three more weeks. Mice were paired-fed, and body weight and food intake were monitored daily. Body weight was measured once a week. Adenovirus injection (2 × 10^9^ pfu) through the tail vein was administered at weeks 2 or 3 of the liquid diet period to suit each experiment. At the end of the experiment, mice were sacrificed and liver tissues and blood samples were collected. Male SIRT1 transgenic mice were purchased from Jackson Laboratory (Bar Harbor, ME). Binge drinking was modeled using a modified version of a recently published protocol^3^. Briefly, twelve-week-old male C57BL/6J mice were fasted for 4 h before receiving two gavages of equivalent calories of ethanol at 3.5 g/kg or dextrin-maltose (DM; MP Biomedicals, Santa Ana, CA). Mice were kept on a heating pad for the duration of the experiment to prevent hypothermia. Then, mice were sacrificed 6 h later. Liver tissues were fixed with 10% (vol/vol) formalin and embedded in paraffin, and were stained with H&E or oil-red-O. All experimental protocols involving animals, including maintenance and care, were performed in accordance with the National Institutes of Health guidelines and ethics guidelines of Yonsei University, and all animal procedures were approved by the Committee on Animal Investigations of Yonsei University.

### RNA isolation and analysis of gene expression by quantitative RT-PCR

Total RNA was isolated using TRIzol reagent (Invitrogen, Carlsbad, CA) according to the manufacturer’s instructions. First-strand cDNA synthesis from 5 μg total RNA was performed using SuperScript III reverse transcriptase (Invitrogen) primed with random hexamer primers. Real-time qPCR was performed using SYBR Green Master mix (Applied Biosystems) with a Step One instrument (Applied Biosystems, Foster City, CA). Expression of Rplp0 was also measured as an invariant control. The primer sequences used in real-time qPCR are as follows: PPARγ, 5′-CTCTGGGAGATTCTCCTGTT-3′, 5′-GGTGGGCCAGAATGGCATCT-3′; SREBP1c, 5′-GGAGCCATGGATTGCACATT-3′, 5′-GGCCCGGGAAGTCACTGT-3′; ChREBP, 5′-CCTCACTTCACTGTGCCTCA-3′, 5′-ACAGGGGTTGTTGTCTCTGG-3′; aP2/422, 5′-TCTCCAGTGAAAACTTCGAT-3′, 5′-TACGCTGATGATCATGTTG-3′; FSP27, 5′-TCCAGGACATCTTGAAACTT-3′, 5′-GGCTTGCAAGTATTCTTCTG T-3′; Cd36, 5′-TGCACCACATATCTACCAAA-3′, 5′-TTGTAACCCCACAAGAGTTC-3′; FAS, 5′-AAGCC GTTGGGAGTGAAAGT-3′, 5′-CAATCTGGATGGCAGTGAGG-3′; MGAT1, 5′-CTGGTTCTGTTTCCCGTTGT-3′, 5′-TGGGTCAAGGCCATCTTAAC-3′; L-PK, 5′-CCGAGATACGCACTGGAGTC-3′, 5′-GTGGTAGTCCACCCACACTG-3′; SCD1, 5′-TTCTCAGAAACACACGCCGA-3′, 5′-AGCTTCTCGGCTTTCAGGTC-3′; GPAT, 5′-TCCTAGCTCGCGATTTCGAC-3′, 5′-ATCTTTCCTGCTCGTGTGGG-3′; Elovl6, 5′-TGCTGATGGGCTGTGTCATT-3′, 5′-GGAGTAGCACTGGTCGTTGT-3′; G0S2, 5′-AAAGTGTGCAGGAGCTGATC-3′, 5′-GGACTGCTGTTCACACGCTT-3′ and Rplp0, 5′-GCAGGTGTTTGACAACGGCA G-3′, 5′-GATGATGGAGTGTGGCACCG A-3′.

### Western blot analysis

For protein preparation from liver tissues, mouse livers (50 mg) were placed in a glass homogenizer containing 1 ml Pro-Prep Protein Extraction Solution (Intron Biotechnology, Korea). Tissue lysates were separated by SDS-PAGE. Primary antibodies against SIRT1, PPARγ (Santa Cruz Biotechnology, Santa Cruz, CA), acetylated lysine (Cell Signaling Technology, Danvers, MA), and β-actin were used. Bands were detected with anti-rabbit or anti-mouse IgG conjugated with horseradish peroxidase (Pierce) using the ECL-PLUS detection system (Amersham, Little Chalfont, UK).

### Primary hepatocyte culture

Primary mouse hepatocytes were isolated using the two-step collagenase perfusion method from the livers of male C57BL/6 (8 weeks old) mice as previously described^35^. Hepatocytes were plated onto six-well dishes at 1.0 × 10^6^ cells per well and incubated for 12 h in DMEM containing 10% FBS to allow cells to attach. Cell counts and viability (Adam cell counter; Digital Bio) were confirmed before use. Viability was routinely >85%. After attachment, cells were infected with PPARγ2- and SIRT1-expressing adenoviruses.

### Preparation of recombinant adenovirus

Murine PPARγ2 and SIRT1 cDNAs were cloned into the pcDNA3 or FLAG-tagged pcDNA3 vectors, respectively. Recombinant adenovirus (Ad) expressing PPARγ2 and ad-shRNA for MGAT1 were prepared as described^15^. All viruses were propagated in 293A cells and purified by CsCl density purification, dissolved in 1x HBSS (Invitrogen), and stored at −70 °C. The multiplicity of infection (MOI) was calculated from viral particle numbers. Recombinant adenovirus containing the GFP gene or Ad-US control RNAi were used as controls.

### TG and cholesterol assay in the liver

Liver extracts (from 0.2 g tissues) were prepared by homogenization in chloroform:methanol (2:1, v/v). TG and cholesterol levels were measured using TG assay or cholesterol assay reagents (ThermoFisher Scientific, Waltham, MA). The TG level was calculated from measurements of the absorbance at 500 nM and expressed as mg TG/g liver wet weight.

### Transfection and luciferase assay

HepG2 cells were transfected with the pGL3-MGATs promoter plasmid^15^,^20^ as indicated or pRL-CMV (Promega, Madison, WI) using Lipofectamine (Invitrogen) according to the manufacturer’s instructions. After 24 h, luciferase activity was measured using the Dual Luciferase Reporter Assay System (Promega) according to the manufacturer’s instructions. Firefly luciferase activities were standardized to Renilla activities.

### NAD^+^/NADH assay

The NAD^+^/NADH ratio was measured using NAD/NADH assay kits from Abcam according to the manufacturer’s instructions. Absorbance was measured at 450 nm using the multi-detection reader (VERSA max, Molecular Devices, Sunnyvale, CA).

### Immunoprecipitation and PPARγ acetylation

For immunoprecipitation, we lysed tissues using a passive lysis buffer (50 mM Tris-HCl, pH 7.4, 1% NP-40, 0.25% sodium deoxycholate, 150 mM NaCl, 1 mM EDTA, and protease inhibitor cocktail). Proteins were immunoprecipitated with anti-PPARγ antibody (Santa Cruz Biotechnology), collected with protein A/G conjugated agarose beads (Santa Cruz Biotechnology), and washed three times with lysis buffer. Acetylation of immunoprecipitated proteins was assessed using anti-acetylated lysine antibody (Cell Signaling Technology). Western blots for PPARγ were also performed to assess the total protein quantity.

### Immunocytochemistry

Primary mouse hepatocytes were isolated from the livers of wildtype or SIRT1 transgenic mice (10 weeks old, male) and then transfected with Ad-GFP or Ad-PPARγ2. At the indicated times, cells were washed with PBS, fixed in 4% formaldehyde for 15 min, permeabilized with 0.2% Triton X-100 for 20 min on ice, and then blocked in 3% bovine serum albumin in PBS for 1 h. Cells were then incubated in a blocking solution containing ADRP antibody (1:200 dilution) for 12 h, followed by fluorescein isothiocyanate-conjugated anti-mouse IgG secondary antibody for 2 h. The cells were mounted in 4′,6-diamidino-2-phenylindole. Cells were then visualized with a Confocal Laser Scanning Microscope (Olympus FV1000).

### Statistical analysis

All data are expressed as the mean ± SD with *n* representing the number of analyzed mice. Statistical significance of observed differences between groups was determined using the unpaired Student’s *t*-test. P < 0.05 was considered as statistically significant.

## Additional Information

**How to cite this article**: Yu, J. H. *et al*. Suppression of PPARγ-mediated monoacylglycerol *O*-acyltransferase 1 expression ameliorates alcoholic hepatic steatosis. *Sci. Rep.* **6**, 29352; doi: 10.1038/srep29352 (2016).

## Supplementary Material

Supplementary Information
