# Supplementary material for: Suppression of PPARγ-mediated monoacylglycerol O-acyltransferase 1 expression ameliorates alcoholic hepatic steatosis
Source: Sci Rep. 2016 Jul 11;6:29352. doi: 10.1038/srep29352 (PMC4941543; doi:10.1038/srep29352)
Supplement: Supplementary Information [file srep29352-s1.pdf]

Supplementary Information

Suppression of PPARγ-mediated monoacylglycerol *O*-acyltransferase 1 expression ameliorates alcoholic hepatic steatosis

Jung Hwan Yu, Su Jin Song, Ara Kim, Yoonjeong Choi, Jo Woon Seok, Hyo Jung Kim, Yoo Jeong Lee, Kwan Sik Lee, Jae-woo Kim

Contents : Supplementary Figures and Legends (Figure S1-S4)

Supplmentary Figure S1

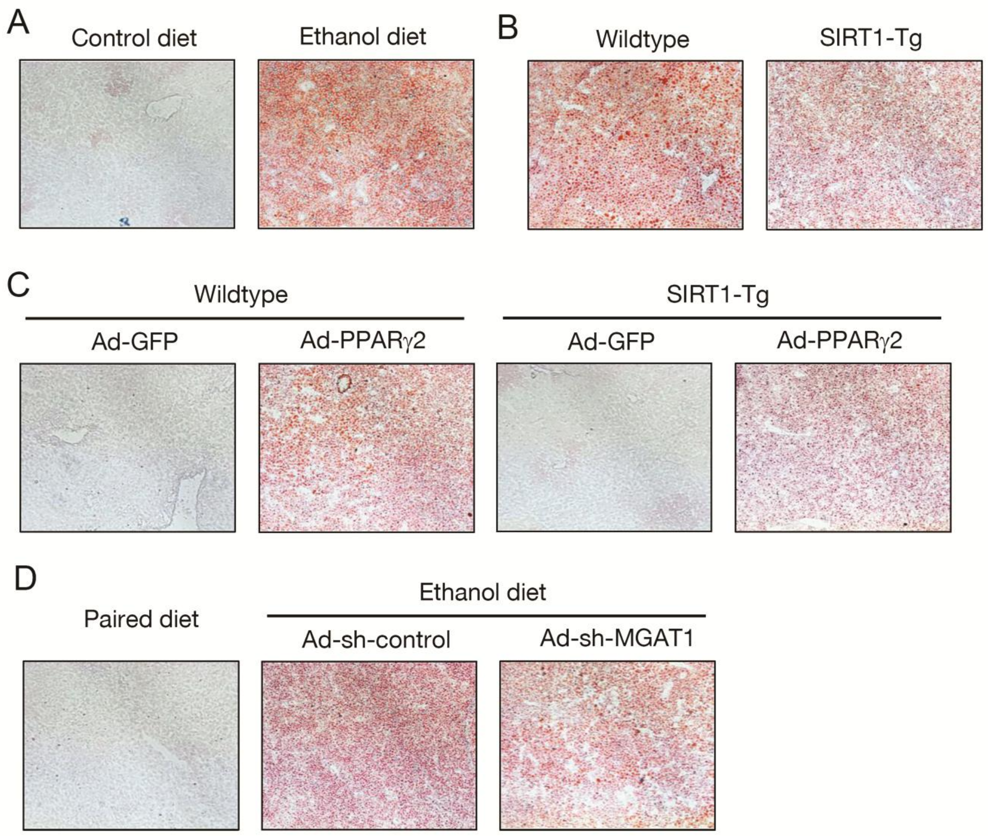

Supplementary Figure S1. Oil-red-O staining performed on liver sections. (A) Oil-red-O staining in control and ethanol diet-fed mice. Related to Fig. 1b. (B) Oil-red-O staining in wild type and SIRT1 transgenic mice after ethanol diet. Related to Fig. 2a. (C) Oil-red-O staining performed on liver sections of wild type and SIRT1 transgenic mice after Ad-GFP or Ad-PPARγ2. Related to Fig. 4b. (D) Oil-red-O staining performed on liver sections from mice in paired, ethanol-fed with sh-control, and ethanol-fed with sh-MGAT1 groups. Related to Fig. 5b.

Supplementary Figure S2

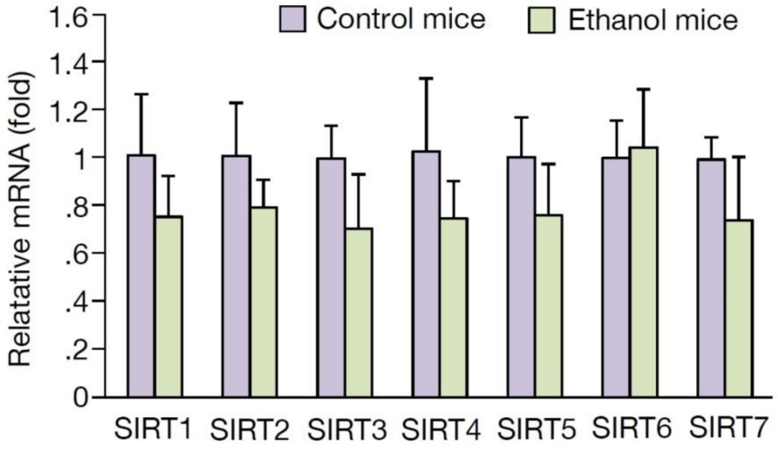

Supplementary Figure S2. mRNA expression levels of sirtuin subtypes in alcohol-fed mice. Expression levels of currently known sirtuin subtypes in the liver of control diet and ethanol-fed mice (n = 6 per group). The mRNA expression levels of all sirtuin types were generally reduced in the ethanol-fed group. Data represent the mean ± SD. *P < 0.05, **P < 0.01.

Supplementary Figure S3

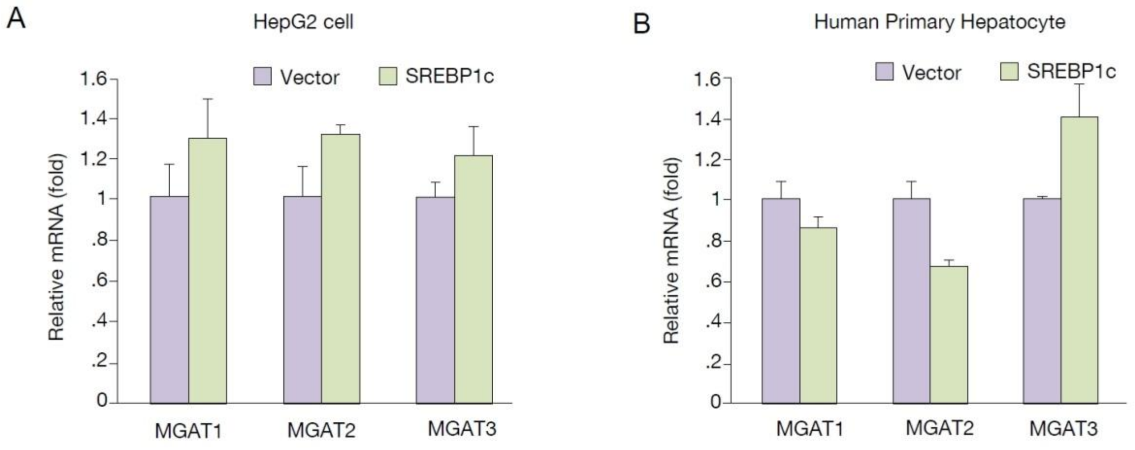

Supplementary Figure S3. Expression patterns of MGAT subtypes in response to SREBP1c overexpression. (A, B) HepG2 cells (4×10^5 cells per well) cultured on 6-well plates or human primary hepatocytes (1×10^6 cells per well) were transfected with SREBP1c expression vector or control vector using Lipofectamine 2000 reagent. After 24 h, the media was replaced with 10% FBS-DMEM. After 48 h, total RNA was isolated and analyzed by real-time qPCR. Data represent the mean ± SD.

Supplementary Figure S4

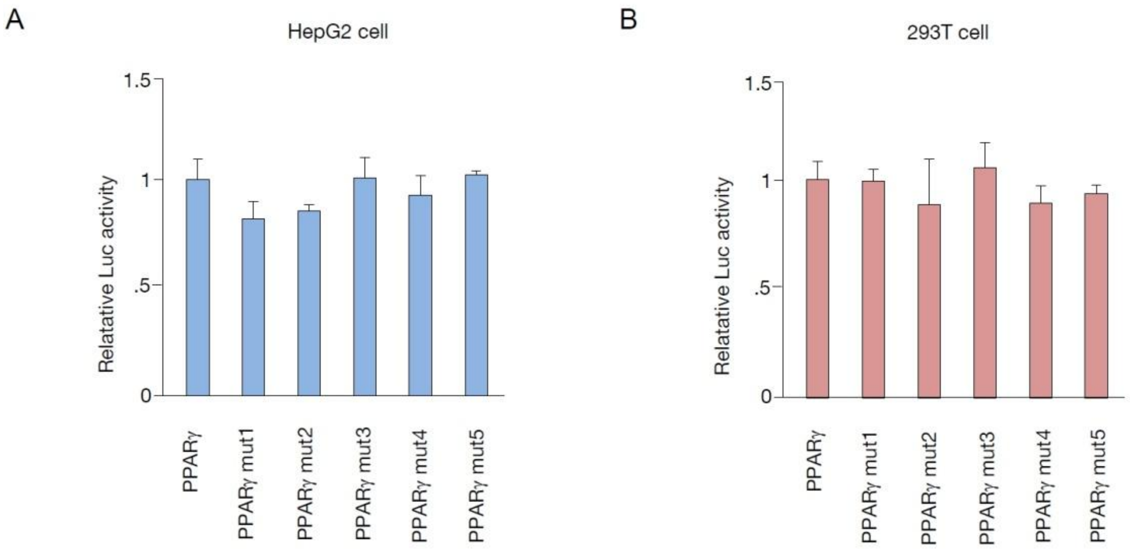

Supplementary Figure S4. Luciferase assay to evaluate the acetylation sites that modulate PPARγ2-induced MGAT1 expression. Luciferase assay using mouse MGAT promoters (~2 kb) was performed. Promoter activity was determined by examining the relative luciferase activity in (A) HepG2 cells or (B) 293T cells. MGAT promoter constructs were co-transfected with each PPARγ2 mutant using Lipofectamine 2000 reagent. Data represent the mean ± SD from three independent experiments performed in duplicate.
